# Necrobiosis Lipoidica in Pregnancy Complicated by Gestational Diabetes Mellitus: A Case Report

**DOI:** 10.7759/cureus.104273

**Published:** 2026-02-26

**Authors:** Mariam Sherif Mohamed, Saba Maqbool, Kashif Rizvi, Panayoti Bachkangi

**Affiliations:** 1 Dermatology, Near East University, Nicosia, CYP; 2 Obstetrics and Gynecology, Lothan Hospital, Kuwait, KWT; 3 Endocrinology, Diabetes, and Metabolism, Lothan Hospital, Kuwait, KWT; 4 Obstetrics and Gynecology, University of Leicester, Leicester, GBR

**Keywords:** gestational diabetes mellitus, granulomatous dermatosis, lower limb dermatoses, necrobiosis lipoidica, pregnancy-associated dermatoses

## Abstract

Necrobiosis lipoidica (NL) is a rare chronic granulomatous skin disease that usually presents as well-defined, yellowish-brown atrophic plaques with telangiectasia on the lower extremities. Its pathogenesis is not fully understood, and it is commonly associated with diabetes mellitus.

A 39-year-old gravida 4, para 3 woman at 36 + 5 weeks’ gestation presented with painful, erythematous to yellow-brown atrophic plaques with mild telangiectasia on the lower limbs and lower abdomen. Her pregnancy was complicated by insulin-requiring gestational diabetes and late-onset pre-eclampsia. Lesions progressively enlarged and ulcerated over several weeks and were later confirmed as NL. She was admitted for optimization of maternal glycemic and blood pressure control and underwent induction of labor, with favorable maternal and fetal outcomes.

NL during pregnancy is exceedingly uncommon, and its clinical features may mimic pregnancy-specific dermatoses, often delaying diagnosis. We report a case of NL in late pregnancy associated with gestational diabetes mellitus (GDM), highlighting an atypical prenatal presentation.

This case highlights the atypical prenatal presentation of NL associated with GDM and the diagnostic challenges of pregnancy-related dermatoses. Recognizing NL in pregnant women with atypical or ulcerative plaques is crucial, and glycemic optimization, along with multidisciplinary management, supports favorable maternal and dermatologic outcomes.

## Introduction

Necrobiosis lipoidica (NL) is a rare, chronic, granulomatous inflammatory dermatosis characterized by well-demarcated, yellow-brown, atrophic plaques that most commonly affect the pretibial regions of the lower limbs [1]. Although the exact incidence of NL in the general population is not well defined, studies among individuals with diabetes mellitus (DM) report a prevalence ranging from 0.3% to 1.2%, with a marked female predominance of approximately 3:1 [1]. The occurrence of NL during pregnancy is exceptionally uncommon, with only isolated case reports described in the literature [2].

NL classically presents as well-defined, yellow-brown, atrophic plaques with telangiectasia on the shins [1]. Lesions may occasionally occur at other sites, including the abdomen, upper limbs, face, or scalp, in 10%-15% of cases [3], and are usually asymptomatic but can become painful if ulcerated or infected [4]. While the condition is often benign, complications develop in approximately 25%-33% of cases, most notably ulceration, which may result in pain, secondary infection, and a significant reduction in quality of life [3,4].

The rarity of NL in pregnancy, coupled with its potential to mimic pregnancy-specific dermatoses, poses diagnostic and management challenges for clinicians.

## Case presentation

A 39-year-old pregnant woman, gravida 4, parity 3, presented at 36 + 5 weeks’ gestation, attended our clinic in August 2024, with painful skin lesions affecting the lower limbs. Her pregnancy was complicated by gestational diabetes mellitus (GDM), requiring insulin therapy, and recently diagnosed mild late-onset preeclampsia, for which antihypertensive treatment was initiated. Her obstetric history included three previous pregnancies, all complicated by GDM, each resulting in induced vaginal deliveries abroad. Glycemic control during the current pregnancy remained challenging despite insulin therapy.

**Figure 1:**
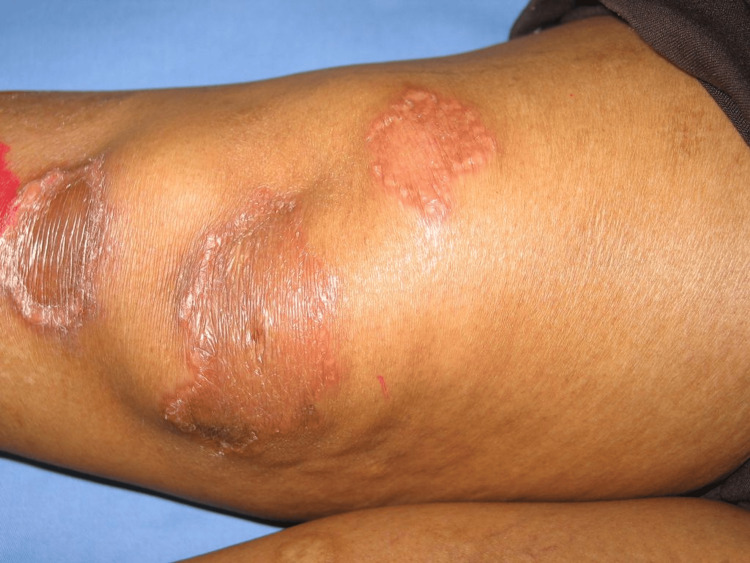
Necrobiosis lipoidica presenting as well-demarcated, erythematous plaques with central atrophy and telangiectasia on the pretibial region. The lesions demonstrate epidermal thinning, waxy appearance, and violaceous borders, consistent with the cutaneous manifestations often associated with diabetes mellitus.

Clinical examination revealed well-defined, erythematous-to-yellow-brown, atrophic plaques with a waxy central appearance and mild telangiectasia on both lower legs (Figure 1). Similar lesions were also observed on the lower abdomen. Most lesions were initially pruritic; over several weeks, several on the lower limbs enlarged and progressed to painful ulceration. Earlier in the pregnancy, the patient had been assessed at other medical centers where the lesions were diagnosed as polymorphic eruption of pregnancy (PEP). Given the atypical morphology, distribution, and evolution of the lesions over time, this diagnosis was reconsidered, and NL was suspected.

On admission, her blood pressure was 160/95 mmHg despite treatment, and her two-hour postprandial glucose level was 9.2 mmol/L, although she remained asymptomatic. Laboratory investigations demonstrated normal renal, liver, and coagulation profiles. However, glycated hemoglobin (HbA1c) was elevated at 7.2% (55 mmol/mol), and urinalysis revealed proteinuria of 3+. The patient reported normal fetal movements. Ultrasound assessment showed an estimated fetal weight at the 90th centile and mild polyhydramnios, with an amniotic fluid index (AFI) of 28 cm.

In view of her complex obstetric comorbidities, including poorly controlled GDM and pre-eclampsia, she was admitted for optimization of blood pressure and glycemic control to ensure maternal and fetal well-being. Following comprehensive counselling, induction of labor was undertaken. Labor and delivery progressed without complication, with satisfactory control of both blood pressure and blood glucose levels throughout her admission.

Active inflammatory skin lesions were managed conservatively with topical corticosteroids, while ulcerated areas received appropriate wound care and dressings. The patient was discharged two days postpartum, with minimal initial improvement in the skin lesions. Subsequent follow-up took place both locally and at other centers, during which a skin biopsy was performed, confirming the diagnosis of NL. By six weeks postpartum, the lesions had completely resolved, coinciding with normalization of glucose metabolism on repeat oral glucose tolerance testing.

## Discussion

NL is rarely reported during pregnancy, and its association with GDM remains poorly understood [5]. Although isolated cases have been mentioned in conference abstracts and small case series [2,5], this case is the first comprehensive, peer-reviewed report describing antenatal presentation, obstetric management, and complete postpartum resolution.

NL is strongly associated with DM, with reported prevalence rates among patients with diabetes ranging from 11% to 65.7%, particularly in the context of suboptimal glycemic control [4]. Pregnancy is associated with profound metabolic, hormonal, and immunological changes, which may exacerbate pre-existing NL or contribute to the development of new lesions [6]. Optimization of glycemic control, therefore, remains central to management [7]. In cases of poorly controlled GDM, NL may persist postpartum, as normalization of glucose metabolism may take several weeks [7].

Topical therapies, including potent corticosteroids and topical tacrolimus, may be effective in non-pregnant patients with NL [8]. For refractory disease, systemic treatments such as corticosteroids, immunomodulatory agents (including Ciclosporin or Thalidomide), and biologic therapies (e.g., TNF-α inhibitors) have been reported [9]. However, the use of these treatments during pregnancy is limited by insufficient safety data, necessitating careful consideration of potential fetal risks [10].

Ulceration is a recognized complication of NL reported in up to 35% of cases and may be associated with pain, infection, and rarely malignant transformation to squamous cell carcinoma [4,11]. Management often requires a multidisciplinary approach involving obstetricians, dermatologists, endocrinologists, and tissue viability specialists [7].

Misdiagnosis remains a significant challenge, particularly in pregnancy [1,2]. A skin biopsy should be considered if the diagnosis is uncertain, the lesion does not respond to treatment, or ulceration is present [2,5].

Diagnostic difficulty is heightened during pregnancy due to the rarity of NL and its resemblance to pregnancy-specific dermatoses (Table 1) [5,12]. PEP, the most common pregnancy-specific dermatosis, occurs in approximately 0.5%-1% of pregnancies and is characterized by intensely pruritic urticarial papules and plaques without atrophy or ulceration [12]. Sparing of the umbilicus is a distinguishing feature. In contrast, pemphigoid gestationis is a rare autoimmune blistering disorder that occurs exclusively during pregnancy, with an incidence of 1 in 10,000-60,000 pregnancies. It is characterized by tense vesicles and bullae and is confirmed by direct immunofluorescence demonstrating linear C3 deposition along the basement membrane [13].

**Table 1:** Differential diagnosis of necrobiosis lipoidica.

Condition	Typical appearance	Primary site	Systemic association	Ulceration risk	Key histopathology	Key differentiating feature	Dermoscopy findings	Reference
Necrobiosis lipoidica	Yellow-brown atrophic plaques with a waxy center and telangiectasia	Pretibial legs	Diabetes mellitus (incl. GDM)	Moderate-high	Palisading granulomas with collagen necrobiosis and lipid deposition	Atrophic plaques with telangiectasia	Yellow-orange structureless areas, linear/branching telangiectasia, whitish scar-like areas	[1,4,12]
PEP	Pruritic urticarial papules/plaques	Abdominal striae, thighs; spares the umbilicus	Pregnancy-specific	None	Non-specific superficial dermal inflammation	Intense pruritus, no atrophy	Non-specific erythema, dotted vessels, no yellow background	[12]
Pemphigoid gestationis	Urticarial plaques → tense bullae	Periumbilical, trunk, limbs	Autoimmune pregnancy disorder	Moderate	Subepidermal blister; linear C3 ± IgG	Positive DIF	Polymorphous vessels, erythematous background, occasional milia-like cysts	[13]
Erythema nodosum	Painful erythematous subcutaneous nodules	Shins	Pregnancy, infection, sarcoidosis	None	Septal panniculitis without vasculitis	Painful nodules without epidermal changes	Poorly defined erythema, blurred vessels	[14]
Morphea	Indurated plaques with ivory center, violaceous border	Trunk, limbs	Autoimmune	Low	Dermal sclerosis, thickened collagen bundles	Skin hardening	Whitish fibrotic areas, reduced vessels, violaceous halo	[20]
Localized scleroderma	Bound-down thickened skin	Limbs/trunk	Autoimmune	Low	Dense dermal fibrosis	Progressive sclerosis	Shiny white areas, decreased follicular openings	[20]
Stasis dermatitis	Eczematous hyperpigmented patches	Lower legs, ankles	Chronic venous insufficiency	Low–moderate	Spongiosis, capillary dilation, hemosiderin	Associated edema	Brown globules, serpentine vessels, scaling	[16]
Granuloma annulare	Annular plaques with raised borders	Hands, feet, extremities	Weak diabetes association	None	Palisading granulomas with mucin	Annular configuration, no atrophy	Yellow-orange areas with peripheral vessels	[15]
Lipodermatosclerosis	Indurated hyperpigmented plaques; leg contour change	Lower legs	Chronic venous insufficiency	Low-moderate	Fibrosing panniculitis	Fibrotic subcutis	White fibrotic areas, brown pigmentation, irregular vessels	[16]
Cutaneous vasculitis	Palpable purpura, necrotic ulcers	Lower extremities	Systemic inflammatory disease	High	Vessel wall inflammation, fibrinoid necrosis	Purpura, necrosis	Purpuric dots, hemorrhagic areas, irregular vessels	[19]
Diabetic dermopathy	Light-brown macules/patches	Shins	Long-standing diabetes	None	Dermal hemosiderin, collagen alteration	Flat asymptomatic macules	Homogeneous light-brown pigmentation, faint network	[17]
Cutaneous sarcoidosis	Papules, plaques, nodules	Face, trunk, extremities	Systemic sarcoidosis	Low	Non-caseating granulomas	Multisystem involvement	Yellow-orange *apple-jelly* areas, linear vessels	[18]

Other important differential diagnoses include erythema nodosum, which typically presents as painful erythematous subcutaneous nodules [14]. Granuloma annulare should also be considered and is characterized by annular plaques with palisading granulomas and mucin deposition [15]. Venous dermatoses, including lipodermatosclerosis and stasis dermatitis, represent additional considerations [16]. Diabetic dermopathy commonly presents as asymptomatic hyperpigmented macules [17], while cutaneous sarcoidosis may manifest with variable papules or plaques and is frequently associated with systemic involvement [18]. By contrast, cutaneous vasculitis usually presents as palpable purpura or necrotic lesions, predominantly involving the lower extremities, and may progress to tissue necrosis [19].

## Conclusions

NL management focuses primarily on optimizing glycemic control, supported by appropriate local dermatological care. This case report highlights the diagnostic difficulties encountered during pregnancy and raises awareness among obstetricians, gynecologists, and other healthcare professionals. Further case reports and shared clinical experiences are needed to enhance understanding of the presentation, management, and outcomes of NL during pregnancy.
